# Attenuation of Post-Shock Increases in Brain Natriuretic Peptide with Post Shock Overdrive Pacing

**Published:** 2010-03-05

**Authors:** Marco Budeus, Emanuel Salibassoglu, Anna Maria Schymura, Nico Reinsch, Nils Lehmann, Heinrich Wieneke, Stefan Sack, Raimund Erbel

**Affiliations:** 1 Department of Cardiology, West-German Heart Centre, University of Duisburg-Essen, Germany; 2 Institute for Medical Informatics, Biometry and Epidemiology, University of Duisburg-Essen, Germany

**Keywords:** brain natriuretic peptide, ICD, predischarge test, heart failure, optimisation

## Abstract

**Background:**

Predischarge defibrillation threshold testing is often performed a few days after ICD implantation in order to validate defibrillation thresholds obtained at the time of implant. Ventricular fibrillation is induced with such testing and causes an increase in serum Brain Natriuretic Peptide (BNP) levels. BNP is an indicator for cardiac stress. We wanted to examine the feasibility to alter the trend of BNP after predischarge testing in VVI, DDD and CRT ICD's.

**Methods:**

We measured BNP before predischarge testing and 5, 10, 20 and 40 minutes after predischarge testing in 13 groups with each 20 patients. We evaluated patients without post shock pacing and patients with a post shock pacing frequency of 60, 70, 80, 90 and 100 bpm and a duration of 30 and 60 sec as well as a post shock pacing frequency of 80 and 90 bpm and a duration of 120 sec post shock pacing.

**Results:**

Patients without post shock pacing showed the highest BNP during the follow-up. The percentage values of BNP increased consistent significantly after 5 minutes compared with BNP before predischarge testing. The percentage values of BNP trend was significantly lower with a post shock pacing of 90 bpm and duration of 60 sec. In addition, we excluded a cardiac necrosis by predischarge testing because of similar values of myoglobin, cardiac troponin I and creatine kinase during the follow-up.

**Conclusions:**

Our results suggested that post shock pacing with 90 bpm and duration of 60 sec as the best optimized post shock pacing frequency and duration for VVI, DDD and CRT ICD's. A reduction of cardiac stress is going to be achieved with the optimization of the post shock pacing frequency and duration.

##  Introduction

The efficacy to treat life threatening ventricular arrhythmias is a generally accepted function of implantable cardioverter defibrillators (ICD's) [1-4]. The tested defibrillation threshold during implantation is usually validated a few days later with predischarge testing [5]. Ventricular fibrillation is induced and terminated for predischarge testing. During the predischarge testing the patients are in acute heart failure [6]. In addition the brain natriuretic peptide (BNP) doubled 5 minutes after predischarge testing as a sign of an acute cardiac tension [7].

Increased ventricular wall tension causes a rapid release of messenger ribonucleic of BNP [8]. Furthermore BNP could reflect the pulmonary capillary wedge pressure and denote rapidly alterations of the cardiac pressure because of the short half-live of 20 minutes [9]. Therefore BNP is a useful neurohormonal marker for the treatment of heart failure [10].

The Dual Chamber and VVI Implantable Defibrillator (DAVID) Trial showed that a long-term ventricular pacing impaired the left ventricular function [11]. But it was unclear whether a short-term ventricular pacing for post shock pacing caused also an impairment.

In our former study we showed a doubling of BNP 5 minutes after predischarge testing with a heterogeneous post shock pacing frequency and duration [7]. The purpose of our prospective study was to alter the trend of BNP after predischarge testing by optimising post shock pacing frequency and duration in VVI, DDD and CRT ICD's.

## Methods

### Study Population and ICD Systems

We included 780 consecutive patients (630 males, 150 female; age 63.5 ± 11.6 years) with VVI-ICD, DDD-ICD or CRT-ICD (260 of each device) during predischarge testing from November 2004 to June 2006. A serum creatinine > 1.5 mg/dl was an exclusion criterion because of possible influence of BNP [12].

The following ICD systems were used: CRT in 260 patients, DDD in 260 patients and VVI in 260 patients. Several ICD's were used: Biotronik Kronos (180), Lexos (3), Lumos (36) and Belos (3); Medtronic Marquis (6), En Trust (201), Sentry (14) and Maximo (6); Guidant Contak (42), Ventak Prizm (1) and Vitality (48); St. Jude Medical Atlas (240). The transvenous endocardial leads were inserted via the cephalic or subclavian vein and positioned under fluoroscopy in subpectoral position in all patients.

### Study Design

There existed different company depended post shock pacing frequencies and durations of the ICD devices. We calculated the lowest common multiple of the post shock pacing frequency as 60, 70, 80, 90 and 100 bpm and of the shock pacing duration of 30 and 60 seconds. We established 10 groups of patients with a homogenous post shock pacing frequency and one group without post shock pacing (nonPSP). We first examined the groups (VVI-ICD, DDD-ICD or CRT-ICD) without post shock pacing. After that we examined the groups (VVI-ICD, DDD-ICD or CRT-ICD) with post shock pacing frequency of 60 bpm and a shock pacing duration of 30 seconds. After completion of each group we examined the next group with increased post shock pacing frequency (60, 70, 80, 90 and 100 bpm). After completion of the shock pacing duration of 30 seconds we repeated this procedure with a shock pacing duration of 60 seconds and further of 120 seconds. We extended the post shock pacing duration to duration of 120 sec in the best two pacing frequency groups. Thus we achieved 13 homogenous groups with a defined post shock pacing frequency and post shock pacing duration. On the basis of our previous study [7] we calculated 20 patients in each group.

BNP (Triage Meter Plus®, Biosite GmbH, Willich, Germany) was measured directly before and 5, 10, 20 and 40 minutes after predischarge testing similar to our previous study [7]. In addition myoglobin, cardiac troponin I and creatine kinase were also measured with the same equipment. The normal values were 0 - 4.3 ng/ml for creatine kinase, 0 - 107 ng/ml for myoglobin and 0 - 0.4 ng/ml for troponin I. The detection limit was 1.0 for creatine kinase, 5 ng/ml for myoglobin and 0.05 ng/ml for troponin I. Blood was taken from a peripheral intravenous catheter.

The local medical ethics committee approved the study protocol and all patients gave written, informed consent before entering the study.

### Predischarge testing

The patients were fasting and received no intravenous diuretic in the morning. We performed a short-term anaesthesia with propofol and midazolam in all patients. Ventricular fibrillation was induced by T wave shock. We defined the duration of ventricular fibrillation as interval between the induction of ventricular fibrillation with T wave shock and the termination of ventricular fibrillation with biphasic shock of the ICD. We monitored (Siemens SC 7000) the patients with ECG Holter before and for a period of 15 minutes after predischarge testing. All patients with CRT-ICD were tested in paced rhythm. Patients with single or dual chamber ICD were tested with intrinsic QRS. All patients with dual chamber ICD were tested with a long AV interval of 300 msec in order to achieve an intrinsic QRS. We included only patients who were successfully defibrillated with the first initial delivered shock energy in order to achieve the same condition for all patients in our study.

### Echocardiography

Using a Phillips ultrasonic device (3.5 MHz; model Sonos 5500, Philips Medical System, Andover, Massachusetts, USA) biplanar left ventricular end-diastolic and end-systolic cavity volumes were calculated with Simpson's rule [12] from paired apical four-chamber and apical long-axis echocardiographic images of a minimum of five cardiac cycles. Biplanar ejection fraction was calculated as: End-diastolic volume - End systolic volume/End-diastolic volume x 100% [13]. Left ventricular systolic and end diastolic diameter of all patients were measured by M-mode and two-dimensional echocardiography.

### Statistics

All data are presented as mean values ± standard deviation. Datasets were tested for normal distribution. Differences between the categorical variables were evaluated for statistical significance using chi-square test or Fisher's exact test, and Student't test for comparing continuous variables excepting BNP value, as its distribution was skew. Here, the Mann-Whitney U test was employed. As a result of the high standard deviation of real BNP values we calculated the percentage alterations of BNP. The BNP value before predischarge testing was equated with 100% for evaluation of percentage alterations. For comparisons of trends of BNP (%), myoglobin, cardiac troponin I and creatine kinase the Bonferroni-Holm test was performed. A measuring of the linear association between two variables was evaluated using Pearson correlation coefficient and all statistical tests were two-tailed. A P value < 0.05 was considered statistically significant. The statistical package used was SPSS 12.0 for Windows.

## Results

All consecutive patients were included in our study. There were no significant differences in baseline clinical values between the 39 groups. The demographic data were presented in Table 1. Most patients belonged to the NYHA II classification (354 patients) and the predominant cardiac rhythm was sinus rhythm. The left ventricular ejection fraction was nearly 30% in the 39 groups (Table 2).  We observed a bundle brunch block in the ECG of 264 patients (Table 3).

### Predischarge testing

The result of the predischarge testing was similar between the groups (Table 4). Ventricular fibrillation was induced in every patient with the first attempt and successfully defibrillated with the initial delivered shock energy. We did not observe proarrhythmic effects of post shock pacing during the monitored interval of 15 minutes. All patients with dual chamber ICD showed an intrinsic QRS pacing during predischarge testing.

### Enzyme trend after predischarge testing 

We observed an increase of BNP after five minutes after predischarge testing (309.6 ± 86.7 vs. 603.3 ± 78.5 pg/ml, P < 0.0001) followed by a decrease compared with BNP before predischarge testing in all patients (Table 5). In addition, the trend of BNP in all patients also showed a significant difference after 10 minutes (309.6 ± 86.7 vs. 492.3 ± 78.5 pg/ml, P < 0.0001), after 20 minutes (309.6 ± 86.7 vs. 394.7 ± 71.1 pg/ml, P < 0.0001) and after 40 minutes (309.6 ± 86.7 vs. 334.3 ± 67.5 pg/ml, P = 0.042). The trend of BNP was significantly different after 5, 10 and 20 minutes and in some cases after 40 minutes in nonPSP (Table 5). The follow-up of BNP in the group with a post shock pacing of 90 bpm and duration of 60 sec was significantly decreased in relation to other 12 groups (Table 5).

**Figure 1:**
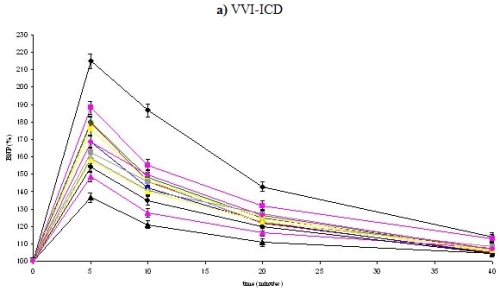

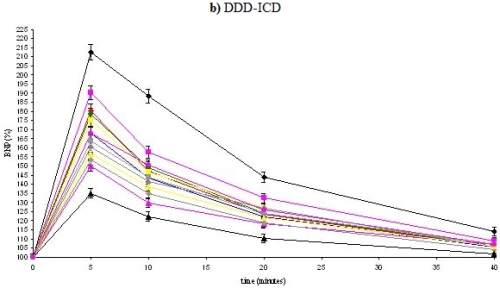

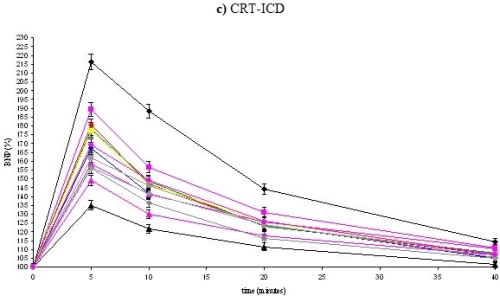
BNP trend in percentage values. ♦ and black line = group NonPSP, ■ and pink line = group 60/30, ♦ and red line = group 60/60, ● and green line = group 70/30, ■ and yellow line = group 70/60, ● and blue line = group 80/30, ▲ and pink line = group 80/60, ♦ and grey line = group 80/120, ● and black line = group 90/30, ▲ and black line = group 90/60, ● and pink line = group 90/120, ▲ and yellow line = group 100/30, ■ and grey line = group 100/60

There was also observed an increase of percentage values of BNP after five minutes followed by a decrease of BNP (Figure 1 a-c). The group with a post shock pacing of 90 bpm and duration of 60 sec had the lowest trend of percentage values of BNP for all ICD devices and a significant difference to the most other groups, which might be attributed to the optimization of the post shock pacing and duration (Table 5). A further increase of shock pacing frequency or duration impaired the trend of BNP (Figure 1 a-c). The highest increase of percentage values of BNP was found in the group without post shock pacing (Figure 1; Table 5).

The trend of the cardiac enzymes (myoglobin, cardiac troponin I, creatine kinase) showed no significant increase after predischarge testing (Table 5).

### Correlation

We observed no correlation between the trend of BNP, myoglobin, cardiac troponin I, creatine kinase or other value (cycle length of ventricular fibrillation, duration of ventricular fibrillation, defibrillation threshold, medication, ICD device, echocardiographic parameters, ECG parameters, age, creatinine). We found a negative correlation between BNP before predischarge testing and left ventricular ejection fraction (r = -0.79, P < 0.0001).

## Discussion

 Our results supported considering the percentage values of BNP an optimized post shock pacing frequency with 90 bpm and duration with 60 sec. Therefore a reduction of cardiac stress was achieved by the optimization of the post shock pacing. In addition, a cardiac ischemia or a correlation between the trends of BNP with other values was excluded.

### Brain natriuretic peptide and ventricular tachycardia

In the present study we showed the well-known increase of BNP after predischarge testing with a following consistent decrease of BNP as a result of ventricle wall tension [7,8,15,16]. This trend could be optimized by modifying post shock pacing with a frequency of 90 bpm and duration of 60 sec, which caused lowest increase of BNP.

Our results were not contrary to the results of DAVID or an analysis of the Multicenter Automatic Defibrillator Implantation Trial (MADIT) II concerning a deterioration of heart failure due to ventricular pacing [11,17]. A frequent right ventricular pacing worsened heart failure but we examined patients with a short-term ventricular pacing after the induction of ventricular fibrillation. Furthermore a worsening of heart failure was caused by histological alterations, which was not expected after short-term pacing [18].

Ventricular fibrillation caused a cardiac low output with an increase of left ventricular end-diastolic pressure and dilation of the right and left ventricle [6,19,20]. BNP and atrial natriuretic peptide increased with a slight temporary delay as a result of the hemodynamic alteration [7-9,15,16]. This increase could significantly decrease due to an optimized post shock pacing in our study. In addition, an influence on the trend of BNP was excluded by other factors with the exception of post shock pacing.

An optimised hemodynamics might improve the clinical outcome with optimal post shock pacing after termination of ventricular arrhythmias. Our results showed an improvement of hemodynamic after termination of ventricular arrhythmias. The present  study examined only a short time period. Prospective trials are needed for the improvement of clinical outcome in patients with optimised post shock pacing.

### Cardiac enzyme trend after predischarge testing 

In the present study we also excluded a myocardial necrosis with similar values of myoglobin, cardiac troponin I and creatine kinase [7]. Other authors observed in different conditions an increase of myoglobin, cardiac troponin I and creatine kinase [21,23]. An increase of cardiac enzyme was related to multiple cardioversions, multiple inductions of ventricular fibrillation or a traumatic injury through the lead implantation [21-23]. These important facts might explain the increase of the cardiac enzymes of the other studies [21-23]. But the sensitivity of the test and the time frame of our measurements could be a reason for similar values of myoglobin, cardiac troponin I and creatine kinase.

## Limitations

We could not exclude a dilution of our BNP values because blood samples were taken from a peripheral intravenous catheter. Furthermore a myocardial necrosis in the further follow-up might be available because an increase of myoglobin, cardiac troponin I and creatine kinase was observed two hours after defibrillation threshold validation [21-23]. We found no troponin I values about the detection limit of 0.05 ng/ml. Thus we could not exclude a significant alteration of troponin I below this detection limit. The programming of the post shock pacing was depending on company device. We calculated the lowest common multiple of the post shock pacing and increased the frequency and duration in dependence on common programs. Thus we could not exclude that a post shock pacing for example with a frequency of 85 bpm or duration of 45 or 75 sec was superior to our results. We did not randomize our groups because we sequentially filled the groups up and the pacing protocol based on the device selection. The clinical outcome of our results is unclear and has to be examined in a larger prospective study.

## Conclusions

We found an optimized post shock pacing frequency of 90 bpm and duration with 60 sec. A reduction of cardiac stress is going to be achieved with the optimization of the post shock pacing frequency and duration. The clinical outcome might be improved by optimized post shock pacing.

## Figures and Tables

**Table 1:**
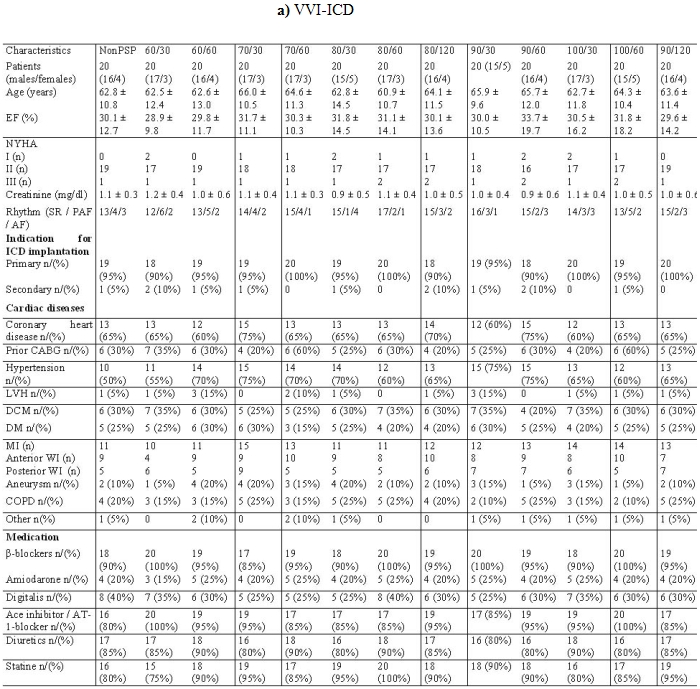

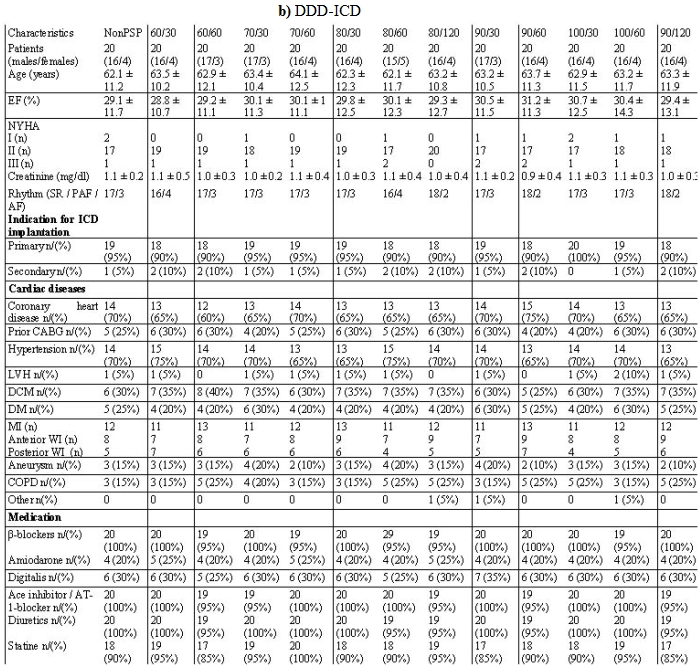

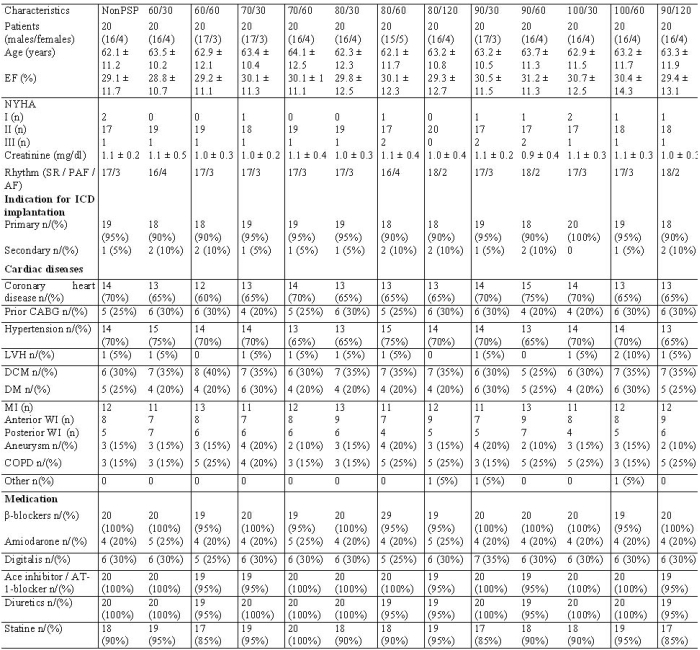
Patients characteristic

Abbreviation: NonPSP: group without post shock pacing, 60/30 = group with a post shock pacing of 60 bpm and a duration of 30 sec, 60/60 = group with a post shock pacing of 60 bpm and a duration of 60 sec, 70/30 = group with a post shock pacing of 70 bpm and a duration of 30 sec, 70/60 = group with a post shock pacing of 70 bpm and a duration of 30 sec, 70/60 = group with a post shock pacing of 70 bpm and a duration of 60 sec, 80/30 = group with a post shock pacing of 80 bpm and a duration of 30 sec, 80/60 = group with a post shock pacing of 80 bpm and a duration of 60 sec, 80/120 = group with a post shock pacing of 80 bpm and a duration of 120 sec, 90/30 = group with a post shock pacing of 90 bpm and a duration of 30 sec, 90/60 = group with a post shock pacing of 90 bpm and a duration of 60 sec, 100/30 = group with a post shock pacing of 100 bpm and a duration of 30 sec, 100/60 = group with a post shock pacing of 100 bpm and a duration of 60 sec, 90/120 = group with a post shock pacing of 90 bpm and a duration of 120 sec.

**Table 2:**
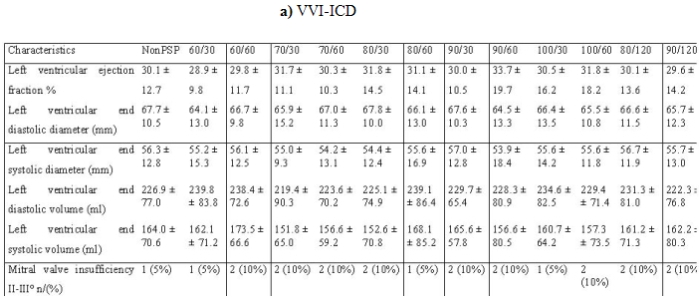

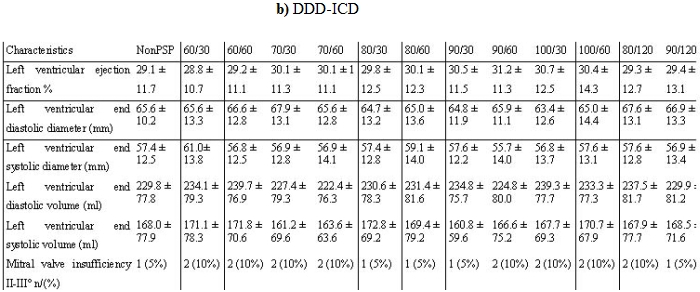

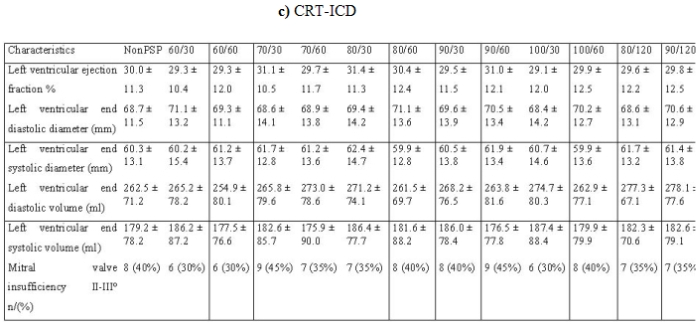
Echocardiographic parameters

**Table 3:**
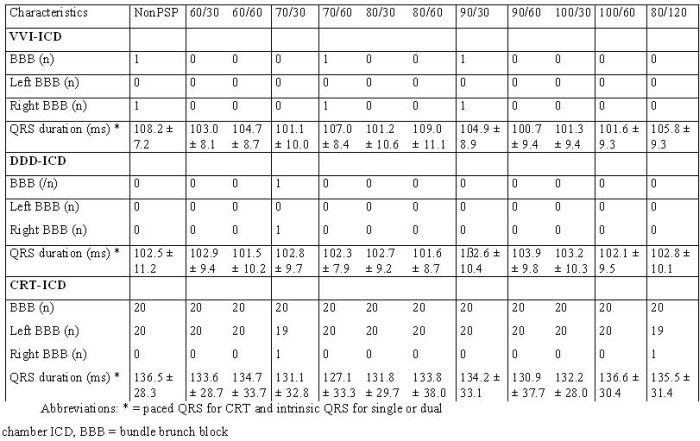
Findings in ECG

**Table 4:**
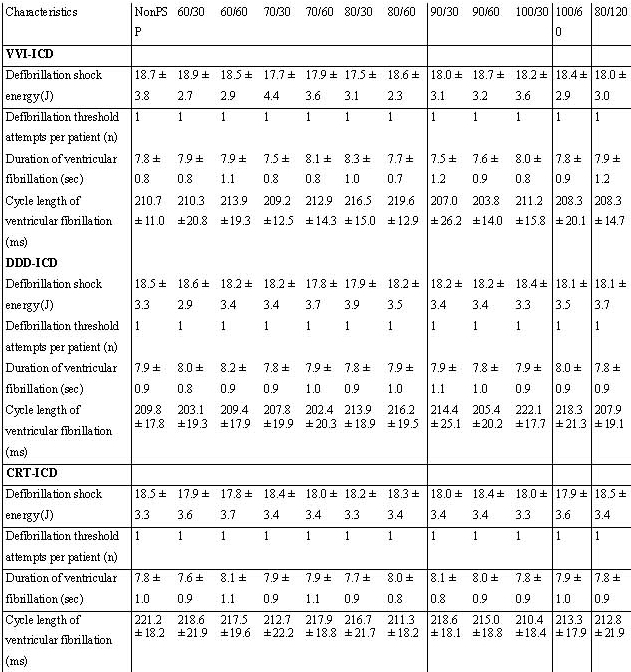
Results of predischarge testing

**Table 5:**
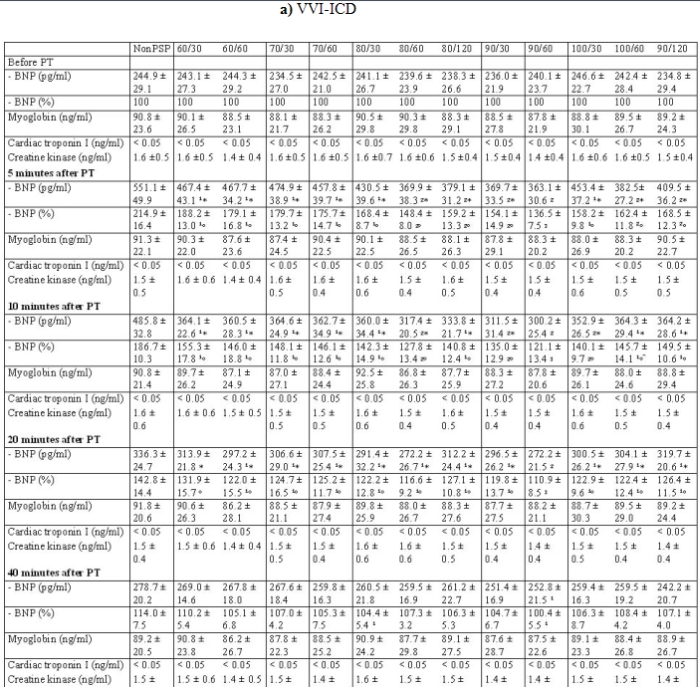

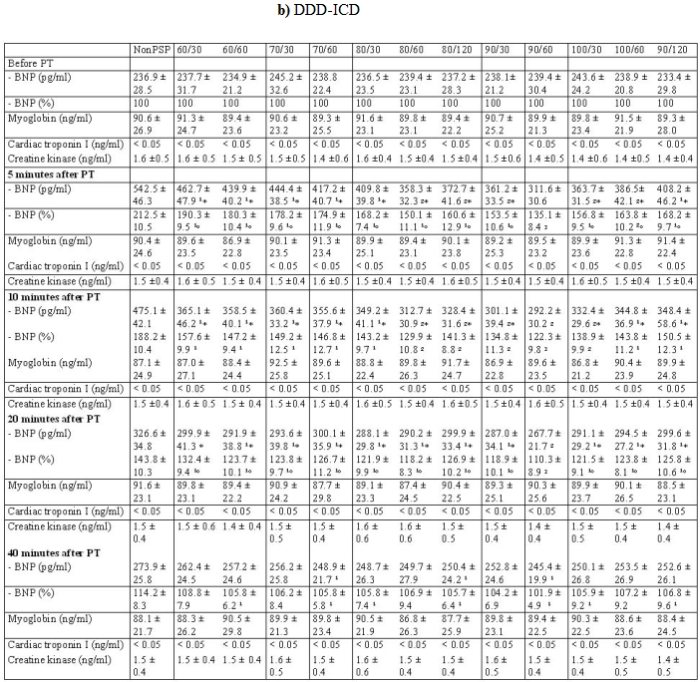

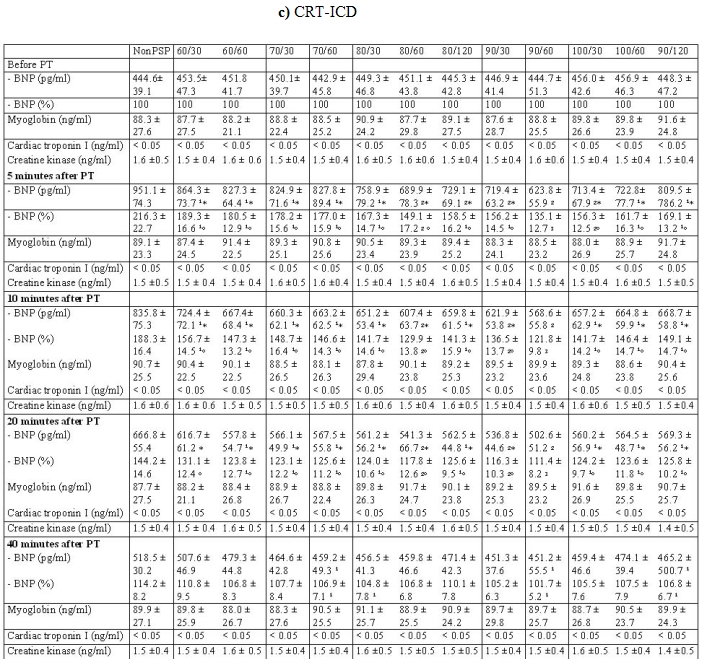
Values of BNP, myoglobin, cardiac troponin I and creatine kinase

Abbreviation: PT = predischarge testing, ^¹^ = P < 0.01 in comparison to group NonPSP, ^²^ = P < 0.005 in comparison to group NonPSP, ^³^ = P < 0.001 in comparison to group NonPSP, * = P < 0.05 in comparison to group 90/60, º = P <  0.03 in comparison to group 90/60.

## References

[R1] Moss AJ (1996). Improved survival with an implanted defibrillator in patients with coronary disease and high risk for ventricular arrhythmia. N Engl J Med.

[R2] Moss AJ (2002). Prophylactic implantation of the defibrillator in patients with myocardial infarction and reduced ejection fraction. N Engl J Med.

[R3] Kadish A (2004). Prophylactic defibrillator implantation in patients with nonischemic dilated cardiomyopathy. N Engl J Med.

[R4] Salukhe TV (2004). Cardiac resynchronisation may reduce all-cause mortality: meta-analysis of preliminary COMPANION data with CONTAK-CD, InSync ICD, MIRACLE and MUSTIC. Int J Cardiol.

[R5] Strickberger SA (1997). Probability of successful defibrillation at multiples of the defibrillation energy requirement in patients with an implantable defibrillator. Circulation.

[R6] Steinbach KK (1994). Hemodynamics during ventricular tachyarrhythmias. Am Heart J.

[R7] Budeus M (2007). Effect of induced ventricular fibrillation and shock delivery on brain natriuretic peptide measured serially following a predischarge ICD test. Indian Pacing and Electrophysiology Journal.

[R8] Cheung BM (1998). Natriuretic peptides-relevance in cardiac disease. JAMA.

[R9] Kazanegra R (2001). A rapid test for B-type natriuretic peptide correlates with falling wedge pressures in patients treated for decompensated heart failure: a pilot study. J Card Fail.

[R10] Troughton RW (2000). Treatment of heart failure guided by plasma aminoterminal brain natriuretic peptide (N-BNP) concentrations. Lancet.

[R11] The DAVID Trial Investigators (2002). Dual Chamber pacing or ventricular buckup pacing in patients with an implantable defibrillator. The Dual Chamber and VVI Implantable Defibrillator (DAVID) Trial. JAMA.

[R12] Tsutamoto T (2006). Relationship between renal function and plasma brain natriuretic peptide in patients with heart failure. J Am Coll Cardiol.

[R13] Schiller NB (1989). Recommendations for quantitation of the left ventricle by two-dimensional echocardiography. American Society of Echocardiography Committee on Standards Subcommittee on Quantitation of Two-Dimensional Echocardiography. J. Am. Soc. Echo.

[R14] St John Sutton M (1998). Quantitation of left ventricular volumes and ejection fraction in post-infarction patients from biplane and single plane two-dimensional echocardiograms. Eur Heart J.

[R15] Fromer M (1998). Effect of induced ventricular tachycardia on atrial natriuretic peptide in humans. J Am Coll Cardiol.

[R16] Cohen TJ (1991). Neuroendocrine response of ventricular tachycardia in humans. Am Heart J.

[R17] Steinberg JS (2005). MADIT II Investigators. The clinical implications of cumulative right ventricular pacing in the multicenter automatic defibrillator trial II. J Cardiovasc Electrophysiol.

[R18] Karpawich PP (1990). Developmental sequelae of fixed-rate ventricular pacing in the immature canine heart: an electrophysiologic, hemodynamic, and histopathologic evaluation. Am Heart J.

[R19] Sylvester E (2003). Defibrillation causes immediate cardiac dilation in humans. J Cardiovasc Electrophysiol.

[R20] Joglar JA (1999). Effects of repeated electrical defibrillations on cardiac troponin I levels. Am J Cardiol.

[R21] Schluter T (2001). Effects of implantable cardioverter defibrillator implantation and shock application on biochemical markers of myocardial damage. Clin Chem.

[R22] Hurst TM (1999). Detection of myocardial injury during transvenous implantation of automatic cardioverter-defibrillators. J Am Coll Cardiol.

[R23] Runsio M (1997). Myocardial injury after electrical therapy for cardiac arrhythmias assessed by troponin-T release. Am J Cardiol.

